# Redefining just cause for contract termination in FIFA's RSTP: striking a fair balance between player rights and club interests in light of the interim regulatory framework

**DOI:** 10.3389/fspor.2026.1845394

**Published:** 2026-08-11

**Authors:** Walid Ben Salah, Nayel AlOmran, Noor Alhendi, Zouhaier Nouri

**Affiliations:** 1 Legal Department, Zayed University, Dubai, United Arab Emirates; 2 United Arab Emirates University College of Law, Al Ain, United Arab Emirates; 3 Public Law Department, University of Tunis El Manar, Tunis, Tunisia

**Keywords:** abusive conduct, contract termination, just cause, outstanding salaries, professional football players, RSTP, sporting just cause

## Abstract

**Introduction:**

This article examines the concept of “just cause” for the unilateral termination of professional football employment contracts under FIFA's Regulations on the Status and Transfer of Players (RSTP). It pays particular attention to the changes introduced by FIFA's 2025 Interim Regulatory Framework. The RSTP aims to protect contractual stability while safeguarding the rights of both players and clubs. However, the meaning and application of “just cause” continue to raise important legal questions.

**Methods:**

The article uses a doctrinal legal approach. It examines the RSTP, the 2025 Interim Regulatory Framework, the FIFA Commentary on the RSTP, decisions of the FIFA Dispute Resolution Chamber (DRC), and awards of the Court of Arbitration for Sport (CAS). It also draws on academic literature and selected comparative sports law studies to examine the development of FIFA's legal framework.

**Results:**

The study shows that the 2025 Interim Regulatory Framework provides useful clarification, especially on the definition of just cause, the burden of proof, and the procedures before the Football Tribunal. Despite these changes, the application of the rules still depends largely on the assessment of each individual case. Important uncertainties remain regarding abusive conduct, financial default, sporting just cause, and the balance between contractual stability and the employment rights of players. The analysis also identifies differences in the approach taken by the FIFA DRC and CAS, which reduce legal certainty and predictability.

**Discussion and conclusion:**

The article concludes that the 2025 reforms are a positive step towards greater legal clarity, but they do not resolve all the weaknesses of the current system. The concept of just cause would benefit from a clearer definition and more objective criteria for its application. Greater consistency in the decisions of FIFA and CAS would also improve legal certainty and strengthen the balance between contractual stability and the legitimate interests of players and clubs. Finally, the article provides one of the first detailed legal analyses of the 2025 Interim Regulatory Framework and offers recommendations to improve the regulation of professional football employment contracts.

## 1 Introduction

The global football industry is governed by a complex regulatory framework, primarily shaped by the Fédération Internationale de Football Association (FIFA), which has exclusive jurisdiction over issues of an international nature. FIFA's control over football matters is extensive, covering international employment contracts, players transfers, the protection of minors (1)^1^, the profession of intermediaries and crucially, the standardization of professional football players’ contracts. In particular, FIFA plays a central role in regulating and overseeing professional football players’ contracts through binding and worldwide-reaching regulations, especially the Regulations on the Status and Transfer of Players (RSTP) (2)^2^.

However, FIFA's dominance over the governance of professional football is no longer absolute. Its regulatory autonomy is increasingly influenced, and sometimes constrained, by external legal forces, particularly European Union law (3)^3^.

Since the landmark Bosman ruling of the Court of Justice of the European Union (4)^4^, EU legal principles have progressively influenced the structure and operation of the international transfer system, leading to significant regulatory adjustments to ensure compliance with the rules of free movement and competition (5)^5^.

This dynamic has continued in recent years, with a more thorough examination of areas such as the regulation of intermediaries and the capping of agents’ commissions (6)^6^. These developments demonstrate that FIFA's regulatory framework does not operate in isolation, but rather within a broader legal environment where EU law plays a structuring role. Consequently, FIFA has had to adapt its rules to reconcile its objective of contractual stability with the legal requirements imposed by supranational standards. In this evolving context, the interpretation and application of key concepts such as “just cause” cannot be fully understood without taking into account this external legal influence, which continues to shape the balance between regulatory autonomy and the protection of economic freedoms in professional football.

Recent developments, particularly the decision of the Court of Justice of the European Union (CJEU) dated October 4, 2024 (Case C-650/22, Lassana Diarra and FIFPRO v. FIFA and URBSFA, 2024)^7^, have brought aspects of the RSTP under scrutiny. The so-called Lassana Diarra case raised several elements of the FIFA Regulations on the Status and Transfer of Players (RSTP) for discussion, in particular, Article 17, which regulates compensation for unilateral termination of contracts without just cause^8^.

In response, while FIFA publicly maintained that the fundamental structure of the international transfer system remains unaffected [such as registration periods, sporting sanctions, training compensation, solidarity mechanisms (7)^9^, and the dispute resolution system], it has nonetheless launched a global stakeholder dialogue^10^ and, subsequently, adopted an Interim Regulatory Framework in December 2024 to address legal uncertainties^11^. This framework introduces key procedural and substantive clarifications, which include a new “just cause” definition.^12^

FIFA's commitment to contractual stability continues to constitute a foundational objective of the RSTP. According to Article 13, a professional contract may only be terminated upon expiry or by mutual agreement. Yet, Article 14 provides an important exception: unilateral termination without consequences is permitted “where there is just cause.”

Indeed, in the presence of a just cause, the party who terminates the contract is exempt from paying compensation or facing sporting sanctions. Conversely, termination without just cause has serious consequences. The terminating party will face the obligation to pay financial damages and the imposition of sports sanctions.

The principle of just cause represents a fundamental balance between two competing imperatives: ensuring stability in professional football relationships and protecting the fundamental rights and welfare of players. On the one hand, contract stability is crucial to the development and maintenance of the integrity of the game. The goal is to ensure that clubs and players respect the contractual terms, therefore, providing a stable and predictable environment in football. This stability is essential to ensuring the smooth running of football leagues around the world. It is also crucial to clubs’ and players’ financial and strategic planning.

On the other hand, the stabilization efforts cannot go beyond the rights of players and clubs. Players can find themselves in vulnerable situations bound by contracts that do not align with their professional ambitions or well-being. Just cause acts as a safeguard that allows them to terminate the employment relationship if the continuation of the contractual relationship becomes impossible or unfair. The acceptance of the possibility to end the contract in case of just cause underlines FIFA's awareness of the brittle, fleeting character of players’ careers. It highlights the need for legal mechanisms that can adapt to changing circumstances.

The adoption of FIFA's Interim Regulatory Framework and the introduction of a revised definition of “just cause” raise critical legal and practical questions. Do these changes materially alter the criteria for applying just cause in a way that not only reflects the evolving realities of modern football but also ensures a fair balance between the interests of players and clubs? Furthermore, do they uphold fundamental principles of justice, proportionality, and legal certainty within the context of global employment relationships in professional football?

To address this question, this article adopts a qualitative doctrinal methodology that combines regulatory analysis, jurisprudential examination, and academic literature. It examines FIFA's Regulations governing contract termination and analyzes relevant case law from the FIFA Dispute Resolution Chamber (DRC) and the Court of Arbitration for Sport (CAS).

This research does not aim to provide an exhaustive analysis of all case law decisions on this subject. Rather, it relies on a sufficiently diverse body of case law to highlight consistent interpretative trends and areas of ambiguity or inconsistency in the application of the concept of just cause. The case law examined includes decisions frequently cited in FIFA Commentary or representative of recurring legal issues related to contract termination. While the analysis primarily focuses on FIFA regulations and CAS/DRC jurisprudence, it also incorporates targeted comparative insights, particularly from Swiss law, which significantly shapes CAS reasoning, as well as, where appropriate, general principles of employment law. Through this analytical framework, the article identifies existing gaps, critically evaluates the recent regulatory reforms, and offers practical recommendations to enhance the balance and equity of the framework for all stakeholders in the football industry.

This article makes four principal contributions. First, it provides one of the first systematic analyses of the 2025 Interim Regulatory Framework, assessing whether the codification of ‘just cause’ meaningfully alters existing jurisprudence. Second, it identifies persistent ambiguities in the application of the concept, particularly regarding abusive conduct, procedural requirements, and financial breaches. Third, it critically evaluates the underlying tension between contractual stability and the protection of players’ rights, situating FIFA's regulatory approach within broader legal principles, including proportionality, legal certainty, and labor protection. Finally, it advances a set of normative proposals aimed at recalibrating the balance between contractual stability and the protection of players’ rights, contributing to ongoing debates on the reform of football's regulatory framework.

The article focuses first on the conditions of just cause as defined by the RSTP and the jurisprudence of the FIFA Dispute Resolution Chamber (DRC) and Court of Arbitration for Sport (CAS) (2). Then, practical cases where just cause is accepted (3) or rejected (4) will be presented.

## 2 FIFA's codification of just cause: jurisprudential continuity and practical ambiguities

With the amendment of Article 14(1) in the RSTP, the Interim Regulatory Framework provides a more comprehensive and codified definition of “just cause”. Until the introduction of the new regulations, just cause had not been defined under the RSTP but was addressed under Article 14, which stated that in the case of just cause, either a player or the club may unilaterally terminate a contract without consequences (meaning compensation payments and sporting sanctions shall not apply). However, this single provision was insufficient to capture the whole meaning of the concept. In practice, its interpretation relied heavily on the FIFA Commentary on the RSTP and the jurisprudence of the FIFA Dispute Resolution Chamber (DRC) and the Court of Arbitration for Sport (CAS). These sources collectively shaped the legal threshold for establishing just cause.

Significantly, while the Interim Regulatory Framework clarifies the definition, it does not alter the substantive conditions required to invoke just cause. The party wishing to terminate the professional player's contract for just cause must ensure that two essential conditions are met. Firstly, the other party must have committed a substantial breach of the contract (2.1). Secondly, termination is generally only possible after complying with a formal notice requirement (2.2).

### 2.1 A substantial breach of the contract

The Interim regulatory framework amended Article 14, paragraph 1 of the RSTP by introducing a broader definition of just cause, aiming to enhance clarity, predictability, and legal security. The newly amended provision now reads:

“1. A contract may be terminated by either party without consequences of any kind (either payment of compensation or imposition of sporting sanctions) where there is just cause. In general, just cause shall exist in any circumstance in which a party can no longer reasonably and in good faith be expected to continue a contractual relationship^13^”.

Although the amendment provides greater clarity, it does not introduce a substantive change to the legal regime already established by FIFA DRC and CAS jurisprudence. Rather, it codifies the existing case-by-case approach and reinforces long-standing interpretations. Also, FIFA hopes that stakeholders who may not be familiar with this procedure will benefit from more clarity and predictability^14^.

According to the Commentary on the RSTP (which is based on CAS jurisprudence^15^), the assessment of the existence of just cause is done on a case-by-case basis, considering the specific circumstances of the case, and applying the following principles:

- Just cause for contract termination by one party exists only when the other party has committed a sufficiently serious violation of their contractual obligations^16^.
- The breach is considered sufficiently serious when objective circumstances would make it unreasonable to expect the working relationship between the parties to continue, such as in cases of a serious breach of trust.
- Contract termination must always be a last resort action (an “ultima ratio” action)^17^, after all other reasonable alternatives have been exhausted.

CAS adopts a restrictive view of just cause that aligns with Swiss law principles. Indeed, the solution embraced in Swiss law is applied by a well-established CAS jurisprudence, which holds that only substantial breaches of a contract can potentially be considered a “just cause” for contract termination^18^.

Thus, in the context of a professional football player's contract and following the principle of contractual stability, unilateral contract termination must be regarded as an absolute last resort when considering the specifics of the situation at hand, it cannot be reasonably expected that either party can continue to be bound by the contractual relationship^19^. Similarly, the jurisprudence of FIFA DRC holds that only a breach or fault of a certain gravity justifies the termination of a professional player's contract. In other words, it is only when there are objective criteria that do not reasonably allow for the continuation of the employment relationship between the parties that a contract can be terminated prematurely. If milder measures can be taken to ensure the other party's compliance with their contractual obligations, these measures must be taken before terminating an employment contract. Premature termination of an employment contract can only ever be an “*ultima ratio*” measure^20^.

The new definition of “just cause” adopted in Article 14, paragraph 1 of the RSTP aims to give football stakeholders greater clarity and legal security. However, it does not introduce any modification of the established jurisprudential principles of FIFA's Football Tribunal, DRC, or CAS jurisprudence. Instead, it codifies existing interpretations.

CAS and FIFA DRC alike have a stringent approach towards just cause, with a preference for contractual stability being an underlying cornerstone. Termination is considered an ultima ratio remedy, which will only be permitted in practice if the party wishing to terminate the contract establishes that:

- The breach is so egregious that proceeding with the contractual arrangement would be objectively unreasonable.
- Less harsh responses, such as warnings and disciplinary procedures, need to be attempted first.
- The termination was a proportionate and final response.

Overall, the revised language in Article 14(1) confirms FIFA's commitment to contractual stability. It reinforces the idea that termination must remain an exceptional remedy, grounded in necessity and proportionality, ensuring contractual stability continues to be a cornerstone of professional football employment relations.

### 2.2 Mandatory default notice

The Interim Regulatory Framework does not modify the notice requirements applicable to contract terminations. Except for Article 14bis, which governs outstanding salaries, the RSTP remains silent on whether such notification is necessary to formally inform the other party of the breach and demand compliance with contractual obligations.

Despite this silence, the Commentary on the RSTP provides interpretative guidance. It attempts to answer whether, in order to establish just cause, it is necessary to first notify the other party of their misconduct or unacceptable behavior before proceeding with termination.^21^ In this regard, the Commentary considers that this notice requirement generally applies. Specifically, when a club seeks to terminate a contract with a player for alleged unjustified absences or improper behavior during training sessions^22^.

Such a notice serves to give the defaulting party a chance to comply with their obligations and, if they deem the request legitimate, to allow them to rectify the situation^23^. According to CAS jurisprudence^24^, for a party to validly terminate an employment contract, they must have formally notified the other party, allowing them the opportunity to comply with their obligations if they believe the complaint is legitimate^25^.

Given this, for a party to validly terminate an employment contract, it is generally necessary to provide the other party with sufficient notice so that they have the opportunity to comply with their obligations if they agree to the just cause. Furthermore, in accordance with the principle of good faith, terminating the contract due to a payment delay is only justified if the employee formally notifies the employer. Before terminating the contract, the employee must first complain that the employer's conduct is not in line with the contract and that they are not willing to accept such violations in the future. The prior formal notice is also a mandatory condition for applying Article 14bis RSTP. Consequently, a player wishing to terminate their contract due to unpaid wages, based on Article 14bis RSTP, must formally notify the club and follow the procedure outlined in this article^26^.

However, the obligation to issue a reminder or formal notice is not an absolute requirement. There are extreme circumstances in which both CAS and FIFA DRC recognize that no reminder or formal notice is necessary when it is evident that the other party has no intention to fulfill their contractual obligations. Under Swiss law, which influences CAS decisions, only a breach of a certain gravity justifies contract termination without notice. In principle, a breach is considered of a certain gravity when objective criteria do not reasonably allow for the continuation of the employment relationship, such as a serious breach of trust^27^. The jurisprudence of FIFA DRC adopts a similar approach. It allows for situations where it is not always necessary to issue a formal notice to the club before terminating the contract due to unpaid wages^28^.

The termination of a professional player's contract is considered effective from the date of sending the formal correspondence containing the contract termination^29^. If such correspondence has not been sent, the date of the filing of the request with FIFA should be considered as the date of contract termination^30^. It's important to emphasize that the unilateral termination of the contract represents a final and irreversible decision that typically cannot be revoked at a later time^31^.

The disciplinary sanction of prematurely terminating a contract is not, in itself, considered just cause. Terminating a professional player's contract remains, as previously discussed, an “ultima ratio” measure. Less severe sanctions are available to the club (warnings, proportionate fines, temporary suspensions, temporary demotion to the reserve team, etc.) and must be exhausted before unilateral termination is considered^32^.

Additionally, the club must provide evidence that the disciplinary procedure has been duly followed and that the player's due process rights have been respected. In the CAS 2017/A/5366 award, the CAS held that a fine imposed by a club on a player should not be enforced if there is no copy of the disciplinary procedure file that should have been applied and if no evidence that this procedure was correctly applied is presented. In other words, proof that the player was able to defend himself and appeal any decision must be established^33^.

In the CAS 2020/A/6854 award, the Panel determined that the disciplinary procedure could not be reopened to impose harsher penalties on the player. Because the club and the player had previously accepted a penalty for his late arrival at the training camp, the club could not (again) punish the player by terminating his contract. Therefore, the premature termination of the employment contract was deemed to have occurred without just cause^34^.

While the revised version of Article 14(1) RSTP provides a definition of just cause, it fails to resolve the ambiguity surrounding the requirement of formal notice in all cases. This lack of clarity and uniformity risks leading to inconsistent decisions, thereby undermining legal certainty for both clubs and players. To enhance fairness and predictability, future amendments to the RSTP should explicitly clarify whether issuing a formal notice is a mandatory prerequisite for valid contract termination.

The Interim Regulatory Framework attempts to bring more clarity to the concept of just cause, but only partially achieves this. The stable case law of sports courts is indeed reflected in them. Still, the use of broad terms and the reliance on discretionary, case-specific analysis make consistent application difficult.

The term “just cause” is broad and may be interpreted differently across cases, leading to unpredictable outcomes and potential inequities. Moreover, these challenges are compounded by the global nature of football, where FIFA's rules must be applied across diverse legal and cultural contexts. Jurisdictions differ in their interpretations of contractual obligations and labor rights, which influence how just cause is understood and enforced.

Therefore, while the codification of just cause in Article 14(1) represents a step toward transparency, it does not eliminate the practical ambiguities or ensure equal protection for all stakeholders. More precise definitions and procedural safeguards are essential to ensure that just cause is applied fairly and consistently in global football. Pending further regulatory clarification, it remains appropriate to rely not only on the regulatory text, but also on the FIFA Commentary on the RSTP and the established jurisprudence of the FIFA DRC and CAS.

In this regard, targeted clarifications could usefully be introduced, without altering the overall structure of the RSTP. In particular, greater precision could be provided on the factors that are already taken into account in practice, such as the seriousness of the breach, the attribution of responsibility, and the relevance of prior notice and the opportunity to remedy the breach, so as to improve the transparency of decision-making. Similarly, further guidance could be developed on the notion of abusive conduct under Article 14(2), for example by identifying illustrative situations drawn from existing case law.

In addition, the role of prior notice could be clarified more explicitly, particularly in cases not governed by Article 14bis. Drawing on the FIFA Commentary and CAS jurisprudence, the regulations could recognize a general expectation that the aggrieved party formally notifies the counterparty of the alleged breach and grants a reasonable opportunity to remedy it, subject to exceptions in cases of particularly serious misconduct. Such clarification would strengthen procedural consistency while preserving the flexibility inherent in case-by-case adjudication.

More broadly, clearer articulation of these procedural expectations would contribute to greater consistency and predictability in the application of the notion of just cause.

## 3 Situations where just cause is accepted

Like other professional sports governing bodies, FIFA imposes rules to limit competition for players’ services. These restrictions aim to regulate player mobility, compensation, and contractual autonomy within and across national leagues. Illustrations of player restrictions include the requirement to compensate previous employers when signing a player. These practices are generally justified by the protection they afford to clubs’ investment in players and to the integrity of sports competitions. However, these restrictions are criticized because they are sometimes excessive and limit competition in the market (8, 9).^35^

It is indeed true that these restrictions often go beyond their intended scope and cause lower player salaries and inefficient distribution of talent across teams. Courts applying competition law have emphasized that such restrictions must be narrowly tailored to pursue legitimate goals such as maintaining competitive balance. Excessive measures, like salary caps or contract restrictions, risk harming players. Such measures reduce competition in the market and limit the possibility for weaker clubs to improve their teams (10).^36^

In order to avoid falling into the pitfalls of competition law, the RSTP attempts to ensure a balance between the need to preserve contractual stability and the protection of players’ rights, while avoiding contravening the general principles of competition law.

In this context and in order to better understand the notion of just cause, it is appropriate to refer to the RSTP, which expressly recognizes specific situations where “just cause” for contract termination is accepted (3.1). Additionally, an analysis of FIFA DRC and CAS jurisprudence provides further insight into other cases where just cause has been recognized, shaping the legal framework governing player contracts while ensuring that restrictive practices do not unduly limit market competition (3.2).

### 3.1 Situations explicitly recognized by the RSTP as just cause

The RSTP explicitly identifies four situations in which just cause for contract termination is explicitly accepted: abusive conduct (3.1.1), the player signing a second employment contract (3.1.2), outstanding salaries (3.1.3), and sporting just cause (3.1.4).

#### 3.1.1 Abusive conduct

The parties to a professional player's contract can be subjected to abuses aimed at coercing them into amending or prematurely terminating contractual terms. It is common for a club to try to force a player to renew their contract by relegating them to the reserve team or depriving them of playing opportunities. Conversely, players may seek to provoke contract termination by deliberately disrupting team harmony, missing training sessions, or refusing to participate in matches under false pretenses.

To put an end to such abusive practices, FIFA made amendments to Article 14 of the Regulations on the Status and Transfer of Players (RSTP) to include a new paragraph regarding abusive situations where one party (either a player or a club) seeks to force the other party to terminate or amend the contract^37^. Thus, Article 14(2) RSTP —adopted before the Interim Regulatory Framework—states, “Any abusive conduct by one party aimed at forcing the other party to terminate or amend the contract entitles the other party (player or club) to terminate the contract for just cause”.

This provision codifies long-standing jurisprudence from both CAS and FIFA DRC^38^, which recognizes that abusive behaviors, whether emanating from the player or the club, constitute just cause for contract termination without consequences.

Article 14(2) RSTP adopts a broad conception of the notion of abuse, leaving considerable discretion to sports tribunals in determining what behaviors are considered abusive. However, the burden of proof lies with the party alleging abuse: players must prove abusive acts by clubs, and clubs must demonstrate abuse by players.^39^

While Article 14(2) remains an essential safeguard against coercive practices in professional football, the burden of proof and the inherently subjective interpretation of what constitutes “abusive conduct” continue to pose challenges. These factors can lead to inconsistent enforcement and legal uncertainty, emphasizing the need for more precise definitions and explicit procedural guidance in future regulatory updates. The Interim Regulatory Framework notably did not address or expand upon the notion of abusive conduct, despite its importance. This omission underscores the necessity for more comprehensive treatment of this issue in subsequent amendments to the RSTP.

In the absence of further regulatory clarification, parties must rely on the FIFA Commentary on the RSTP, as well as relevant jurisprudence from the Court of Arbitration for Sport (CAS) and the FIFA Dispute Resolution Chamber (DRC), to interpret and apply Article 14(2). These sources offer valuable insights into what constitutes abusive behavior, providing examples committed by both clubs and players. Understanding these precedents is critical to navigating the legal framework surrounding just cause in cases of alleged abuse.

- Abusive behaviors committed by clubs

The practice of a club pressuring a player to renew their contract, modify its financial terms, or terminate it by depriving the player of the opportunity to train with the first team is considered misconduct. Indeed, it constitutes an abuse for the club to separate the player from the rest of the team and have them train alone, potentially at inconvenient hours and without the supervision of the coaching staff^40^. Furthermore, a consistent and established jurisprudence of the CAS holds that since football is a team sport, the majority of training should occur within a team or group and involve training with a ball^41^.

The duration of the player's exclusion from the team can be a criterion to determine whether the player's sidelining can be accepted or, on the contrary, constitutes just cause for contract termination^42^. For instance, in the CAS 2014/A/3643 award^43^, CAS ruled that an eight-day team exclusion did not meet the criteria for a just cause for contract termination. Conversely, in the CAS 2013/A/3074 award^44^, CAS deemed that an exclusion lasting more than one month could justify a player's contract termination for just cause. Similarly, in the CAS 2019/A/3074 award, CAS concluded that a “relatively short” exclusion from collective training sessions with the first team (in this instance, lasting 32 days) that was imposed without a specific reason constituted a valid reason for the player involved to terminate their contract with the club^45^.

The exclusion duration is not the only factor CAS considers when determining if sidelining the player is wrongful. CAS relied on several other elements inherent to the relationship between the player and the club. In this case, the following behaviors were considered abusive:

− Preventing the player from participating in any club activities outside of training sessions and matches (e.g., public events, social media appearances)^46^.
− Reducing the player's access to physiotherapy and medical services.
− Depriving the player of certain benefits, such as forcing them to leave their apartment or taking away their car ^47^.

CAS jurisprudence certainly provides valuable guidance on what constitutes abusive conduct. However, the lack of precise regulatory standards creates room for subjective interpretation. Clubs may exploit this ambiguity by engaging in subtle forms of coercion that fall short of clear-cut exclusion but still undermine the player's status and well-being.

For example, a club could allow a player to train with the first team but limit his inclusion in practice matches or actual game-time scenarios. The club could also limit the player's training by allowing him to train alone or with a group of undesirable players. The player may also be ignored by the club's technical and/or medical staff, who would cease or reduce communication with him. Several other subtle practices can also contribute to the social isolation of the player inside the team.

Although the player continues to be part of the first team, his professional development stagnates and his market value declines, which creates indirect pressure on the player, pushing him to terminate his contract or accept a transfer, or, in other situations, to agree to a contract renewal. Although these practices, when combined, can harm a player's career progression, they would likely not be considered abuse within the meaning of Article 14(2). The club would then not be held liable. Furthermore, basing the assessment solely on the duration of the exclusion risks minimizing the negative impact that a series of minor cumulative abuses can have.

In any case, the absence of precise criteria or a regulatory definition of the concept of abuse can lead to an uneven application that varies from one case to another. Future amendments to the RSTP should focus on defining the concept of abuse and establishing clear criteria to distinguish abusive behaviors. Thus, providing legal certainty for clubs and players, and allowing for uniform application of the RSTP rules across cases.

ii. Abusive conduct committed by players

Players can also engage in abusive behavior, intending to force the club to transfer the player or to accept an early contract termination. Although Article 14(2) RSTP does not describe such behaviours, the Commentary on the RSTP cites some examples of abusive behaviors committed by players:

- The player may refuse to train or participate in matches, citing false excuses.
- The player may adopt persistent misconduct such as provoking disputes with teammates, not following the coach's directives, etc.^48^.
- The player's attitude has been characterized by a lack of motivation, conflict seeking, and repeated absenteeism from training sessions without justification.

If it persists, this type of abusive behavior may justify termination of the contract for just cause based on Article 14(2) of the RSTP. However, in such situations, the club's response must be gradual and progressive, beginning with a reprimand or fine. Only if the player's abusive behavior continues despite prior disciplinary sanctions is termination for just cause justified.^49^

Indeed, the Commentary on the RSTP provides valuable clarification that helps better understand the concept of abuse within the meaning of Article 14(2). However, as useful as they may be, these clarifications do not completely dispel doubts. This concept remains subjective, and its interpretation is left to the discretion of sports courts. Furthermore, the Interim Regulatory Framework does not take the trouble to clarify the meaning of abuse and does not codify the criteria for its determination.

This leaves clubs and players uncertain and vulnerable to inconsistent application of this provision. The management of similar situations is particularly complicated for clubs because players constitute economic assets. Indeed, unilaterally terminating a player's contract is a delicate decision to take. Such a decision can not only imply paying damages and risking sports sanctions. It can also involve significant financial losses regarding transfer fees or unamortized investments.

Clubs, therefore, face a dilemma. Should they enforce discipline within the team and terminate the players’ contract, or should they refrain from sanctioning him, tolerate his behavior, and preserve asset value?

Clubs use specific contractual clauses (11)^50^ outlining disciplinary procedures and unacceptable behaviors (like training attendance or adherence to rules of conduct) to fill regulatory gaps. For instance, gross misconduct, noncompliance with a disciplinary warning, or a criminal conviction (12)^51^ are grounds for termination under the standard (13)^52^ Premier League contracts (12).^53^

Ultimately, while FIFA's framework provides a basic structure, it falls short in offering detailed, universally applicable standards for abusive conduct. Clubs must rely on carefully drafted contracts to address this gap, ensuring their rights are enforceable while minimizing exposure to financial and legal risks. A clearer regulatory framework and robust contractual protections are essential for maintaining discipline and economic stability in professional football.

#### 3.1.2 Signature by the player of more than one contract covering the same period

A football player is prohibited from entering into more than one employment contract covering the same period, as stipulated by Article 18.5 of the FIFA RSTP^54^. Violation of this rule, such as a player signing multiple contracts for the same period, invokes sanctions outlined in Article 17 RSTP. Consistent jurisprudence from the Court of Arbitration for Sport (CAS) and FIFA's Dispute Resolution Chamber (DRC) has affirmed that signing a second overlapping contract effectively constitutes a de facto termination of the first contract by the player. This principle is grounded in the need to uphold contractual stability and prevent legal disputes in the sport ^55^.

However, there is a notable exception to this rule in the case of loan agreements. Article 10 RSTP permits the loan of a professional player to another club, provided there is a written agreement that binds the player and both clubs involved. These loan agreements are distinct from standard contracts as they temporarily transfer a player's registration to the receiving club while maintaining the original contract with the lending club. Understanding the nuances of this exception is crucial, as it ensures flexibility in player movement while respecting the integrity of contractual agreements. ^56^

#### 3.1.3 Terminating a contract with just cause for outstanding salaries

Article 14bis RSTP, titled “Terminating a contract with just cause for outstanding salaries” provides in its first paragraph the possibility for a player to terminate their contract in the case of unpaid salaries^57^.

The non-payment of salaries is the most commonly invoked reason by players to end the contractual relationship with the club. This is not surprising since the primary obligation placed on the employing club is to pay the player's wages^58^.

Two cumulative prerequisites must be established for the player to terminate the contract for just cause based on Article 14bis RSTP: The club unlawfully fails to pay a player at least two monthly salaries on their due dates (i), and the player has put the debtor club in default in writing (ii).

- The player can terminate their contract when the club unlawfully fails to pay them at least two monthly salaries on their due dates. The second paragraph of Article 14bis RSTP specifies that for any salaries of a player which are not due on a monthly basis, the pro-rata value corresponding to two months shall be considered. Delayed payment of an amount which is equal to at least two months shall also be deemed a just cause for the player to terminate his contract, provided that the player complies with the condition of issuing a formal notice. The CAS has determined that the calculation of the value corresponding to two months of salaries as stipulated in Article 14bis should be based on the total duration of the contract, rather than the duration of the sporting season.^59^
- The player who wishes to terminate their contract due to non-payment of at least two salaries must follow the procedure outlined in the first paragraph of Article 14 bis RSTP. Therefore, the player must formally notify in writing the indebted club and grant them at least fifteen (15) days to make the full payment of the amounts owed to them. Once the formal notice is received, the club must fulfill all of its financial obligations within the specified time frame if it does not want the contract to be terminated for just cause. Partial payment of the outstanding amounts is not accepted (14)^60^. Furthermore, payments made after the termination of the employment contract will not be considered by the FIFA DRC when determining whether the player terminated the contract with or without just cause (14)^61^.

When both prerequisites are met, the DRC considers that the player has just cause justifying the premature termination of the contract, based on Article 14 bis. However, if either of the two conditions is not fulfilled, Article 14 bis does not apply, and the DRC may decide that there was no just cause for the contract's termination.

While Article 14bis RSTP provides a crucial safeguard for players against unpaid salaries, its rigid application may unfairly impact clubs, particularly those facing financial difficulties. The provision requires that a player must be unpaid for at least two months and issue a formal notice before terminating their contract for just cause. While this aims to ensure contractual fairness, it fails to account for the financial constraints that clubs, especially smaller or less wealthy ones, often experience due to cash flow issues, economic crises, or delayed revenues from broadcasting and sponsorship deals.

One major shortcoming of Article 14bis lies in its failure to distinguish between salaries and bonuses^62^. In practice, a single unpaid bonus may exceed two months’ wages, meaning a player could legally terminate their contract despite having received their regular salary. Since bonuses are typically tied to performance, revenue, or competition outcomes, they should not be treated as equivalent to fixed salary obligations. Clubs often base their budgets on variable income streams, and delays in bonus payments may arise from external factors like late sponsorship or broadcast payments.

This uniform treatment creates a potential loophole: players could terminate contracts based on delayed bonuses, even if the club is not in breach of salary obligations. Compared to standard employment contracts, where missed payments do not automatically justify termination, Article 14bis imposes an unusually strict rule that may disproportionately affect smaller clubs or clubs facing financial difficulties.

A more equitable approach would distinguish between salary and bonus defaults, consider a club's temporary financial hardship, and introduce mechanisms like payment plans or arbitration before allowing contract termination. While protecting players is vital, the current rule is overly rigid. Reforming Article 14bis to introduce nuance and flexibility would help preserve both players’ rights and the financial stability of clubs.

#### 3.1.4 Sporting just cause

Article 15 RSTP allows the “established professional player” to rely on sporting reasons to justify the premature termination of the contract with the club. In cases where sporting just cause is established, no sporting sanctions will be imposed on the player, but a financial compensation may be owed to the club. Article 15 RSTP grants the player an exceptional right to terminate their contract without any fault or negligence being attributed to their club.

That being said, the player's ability to invoke sporting just cause is subject to the fulfillment of several conditions, the existence of which must be strictly assessed and interpreted. Thus, termination for sporting just cause requires the verification of two cumulative conditions (i) and compliance with a specific procedure (ii).

- The player’s ability to terminate their contract for sporting just cause is contingent upon the satisfaction of two cumulative conditions: the player is an established professional player, and the player has participated in fewer than 10% of the club’s official matches. The Commentary refers to a decision of the FIFA DRC ^63^ to establish four objective criteria for determining if a player is an “established professional": the player’s age, their past performance, whether the player’s training period was completed, and the experience of their teammates.
- An established professional player who wishes to terminate their contract based on sporting just cause can only do so during a specific period. They can terminate their contract on this basis only within fifteen days following the club’s last official match of the season. Before the expiration of this deadline, the player must notify the termination of their contract in writing and specify that they are relying on sporting just cause to terminate their contract.

Article 15 of the RSTP stipulates that in the event of the establishment of sporting just cause by the competent body (the FIFA DRC in the first instance and the CAS on appeal), no sporting sanctions are imposed on the player who has terminated the contract. However, the text of the provision specifies that financial compensation is owed to the club.

While Article 15 of the RSTP grants players the right to terminate contracts for sporting just cause, its strict conditions and procedural limitations render it largely ineffective. A player might regularly train with the team yet not meet the threshold due to minimal game time. The requirement to terminate within 15 days after the season ends adds further impracticality, offering limited time for decision-making.

Although Article 15 of the RSTP exempts players from sporting sanctions when terminating a contract for sporting just cause, it still allows for the possibility of financial compensation being awarded to the club. However, the criteria for assessing this compensation remain vague and undefined. This legal ambiguity diminishes the provision's effectiveness and creates uncertainty for both parties. Without transparent guidelines, clubs and players are left unable to accurately predict the legal and financial consequences of invoking sporting just cause, potentially discouraging its use and undermining the rule's intended protective function.

Given that this rule has never been applied in practice, it suggests a fundamental flaw in its design. A more practical approach would involve greater flexibility in match participation criteria, an extended termination window, and a clearer compensation structure, ensuring fairness for both players and clubs while preserving contractual stability.

### 3.2 Situations recognized by the jurisprudence

The combined jurisprudence of FIFA and the Court of Arbitration for Sport (CAS) constitutes the foundation of global football law. This jurisprudence shapes critical aspects such as contract termination, clubs’ obligations, and the protection of player rights (9)^64^. Their rulings carry authoritative weight and establish precedents that directly influence player-club relations across jurisdictions. While the RSTP outlines specific scenarios in which just cause may be invoked, a broader analysis of decisions from the FIFA Dispute Resolution Chamber (DRC) and CAS reveals additional practical contexts where just cause has been recognized. Notably, these include cases involving a club's failure to register a player (3.2.1) and the failure to secure a visa or residence permit necessary for the player to fulfill contractual obligations (3.2.2).

#### 3.2.1 Deregistration or non-registration of a player

A player can only participate in organized football if they are electronically registered^65^. Thus, the player can only carry out their activity and participate in official competitions if their club fulfills the necessary registration formalities. Since registration is a formality and an obligation imposed on the club, the deregistration or non-registration of a player constitutes a violation of the player's fundamental rights attributable to the club.

The jurisprudence of the CAS and the FIFA DRC considers that the deregistration or non-registration of a player constitutes a fault on the part of the club. These situations often arise when a club has already used up its quota of foreign players but wishes to register another foreign player. Since it has already exhausted its quota, the club proceeds to deregister a foreign player it wishes to replace with a new foreign player, without terminating the contract of the deregistered player.

By deregistering a player, even for a limited period, a club effectively prohibits a player's potential access to competition and, as such, violates one of their fundamental rights as a football player. Therefore, the deregistration of a player could, in principle, constitute a contract termination since it effectively prevents a player from being eligible to play for their club^66^.

The CAS largely aligns with the conception adopted by the FIFA DRC. In the award TAS 2016/A/4509, the Panel determined that the player had the right to end his employment contract prematurely. This was because the club that signed him failed to complete the necessary registration procedures in two registration periods. This failure occurred because the club had already reached its limit of three foreign players, to which they were entitled^67^. Likewise, in CAS 2014/A/3643, the Panel held that the deregistration of a player to participate in a national championship gives the player the right to unilaterally terminate their contract for just cause, without the need to send a formal notice to the club^68^. In the award CAS 2015/A/4122, the Sole Arbitrator adopted an even stronger stance as he considered that the de-registration of a player in the Transfer Matching System (TMS) is a factual termination of the employment contract^69^.

#### 3.2.2 Failure to provide a visa or work permit

Obtaining a valid visa or work permit for the player is an obligation incumbent upon the club (15)^70^. A well-established jurisprudence holds that the club is responsible for timely obtaining these documents^71^. Consequently, a player to whom these documents are not timely provided has the right to terminate the contract for just cause. That being said, the player must cooperate in the process of obtaining these documents. Likewise, since the termination of a professional player's contract must be a last resort measure, the player must send a formal notice to the club before ending the contractual relationship^72^.

Jurisprudence unanimously agrees that the club's failure to establish a work permit for its player constitutes just cause on which the player can rely to terminate their contract. In its decision no. 20-00471 dated 6/4/2020, the FIFA DRC considered that obtaining a work permit or the implementation of any other administrative procedure allowing the player to work in the country of the employing club is an obligation incumbent upon the club^73^. In another decision, the DRC considered that the club should inform the player of the extension of the visa. Otherwise, this is considered a breach by the club of its obligations^74^.

## 4 Situations where just cause is rejected

As previously mentioned, only a serious breach or fault justifies the termination of a contract. The premature termination of a professional player's contract can only be a measure of last resort. In this regard, the following causes are not accepted by the FIFA DRC and/or the CAS: Brief absences (4.1), poor performance (4.2), a pandemic (4.3), or player injuries (4.3) are not considered cases of just cause.

### 4.1 A brief absence

A brief absence cannot be considered just cause justifying the premature termination of a professional player's contract. In its decision of November 19, 2020, the FIFA DRC considered that in light of the “ultima ratio” criterion, even if the player had been absent for a few days, such absences could not justify an early termination of the contract, especially as they occurred during a period that traditionally overlaps with the winter break. In the FIFA DRC's view, the club could have taken more lenient measures before unilaterally terminating the contract. Therefore, in line with established jurisprudence in similar cases, the FIFA DRC unanimously concluded that the club had terminated the contract without just cause and that the club should be held responsible for the premature termination of the contract^75^. Additionally, proof of the player's absence must be established by the club^76^.

In another decision, the FIFA DRC held that a club seeking to terminate a player's contract for leaving the club without permission or failing to return after the expiration of authorized leave must adhere to the principle of “ultima ratio.” Less severe disciplinary measures must be imposed first (warnings, temporary suspensions, fines, etc.). Additionally, the club must, before terminating the contract, formally notify the player and instruct them to return to the club within a reasonable period^77^.

### 4.2 Termination for poor performance

Consistent jurisprudence from the FIFA DRC holds that a player's poor performance cannot be invoked as just cause for terminating the contract. In a 2020 decision, the DRC considered that a player's mediocre performance could not constitute just cause. As a result, the club was held responsible for the contract's premature termination without just cause^78^.

Established jurisprudence from the CAS confirms this position. A player's poor sporting performance does not constitute just cause for a player to unilaterally terminate an employment contract, even if there is a clause stipulating this possibility in the contract^79^. According to the Commentary on the RSTP, the DRC and PSC generally maintain that a party cannot be granted the right to unilaterally terminate a contract for just cause when the existence of the relevant circumstances is based solely on that party's own subjective judgment. In other words, just cause must be established through objective criteria, not merely the unilateral perception of, for instance, a club seeking early termination^80^.

FIFA DRC and CAS jurisprudence consistently rejects the notion that poor performance constitutes just cause for contract termination, thereby preserving strong job security for professional players. While this position would rightly aim to protect players from arbitrary or subjective dismissals, it could overlook the legitimate concerns of clubs faced with players exhibiting ongoing negligence, a lack of motivation, or maintaining an unprofessional lifestyle, such as excessive weight gain or poor attention to fitness and hygiene. This strict interpretation would place clubs in a difficult position, requiring them to uphold contracts even when a player's conduct seriously undermines team performance and professional standards.

In contrast, in most employment sectors outside of football, sustained underperformance could justify dismissal, provided objective evaluation criteria and fair procedures are followed^81^. Certainly, the specificity of professional football justifies a more cautious approach. The restrictive position adopted by FIFA DRC and CAS is rooted in the principles of contractual stability and legal certainty, as well as the need to protect players from subjective or potentially discriminatory performance assessments. Performance in football is inherently multifactorial, shaped by team dynamics, tactical decisions, injuries, and other external variables, which makes it difficult to isolate individual responsibility with sufficient objectivity. From this perspective, the current jurisprudence seeks to prevent opportunistic or abusive terminations and to preserve the integrity of contractual relationships.

Nevertheless, this strict approach may place clubs in a structurally disadvantaged position, requiring them to bear the financial consequences of sustained underperformance even in cases where the player's conduct significantly undermines professional standards. Furthermore, even where performance-based clauses are contractually agreed upon, CAS jurisprudence tends to regard them as unenforceable, thereby limiting clubs’ ability to ensure accountability through contractual mechanisms.

Against this background, a carefully calibrated reform could be envisaged. Such an approach would allow narrowly tailored contractual provisions addressing severe and sustained underperformance, provided that they are based on objective evaluation processes and robust procedural safeguards. This would help reconcile the protection of players against arbitrary decisions with the legitimate interest of clubs in maintaining professional standards and competitive performance.

### 4.3 COVID-19 pandemic

The COVID-19 pandemic does not constitute just cause for the termination of a professional player's contract. In a decision dated October 22, 2020, the FIFA DRC concluded that a termination due to the COVID-19 pandemic would be considered a termination without just cause^82^. In another decision, the FIFA DRC noted that, according to the document “COVID-19: Regulatory Issues in Football,”^83^ FIFA had not declared the COVID-19 outbreak as a case of force majeure in any country or territory. It also did not state that any employment contract or specific transfer should be affected by force majeure^84^.

Consistent jurisprudence from the CAS holds that the COVID-19 pandemic is not a justification for clubs to unilaterally reduce (without the player's agreement) or cease paying player salaries^85^. Likewise, the pandemic does not constitute a force majeure that allows a player to terminate their contract. In the CAS 2021/A/7851 & 7905 award, the CAS concluded that the player could not rely on the pandemic to terminate his contract, even if (i) the player could not participate in football matches because the competition was suspended in Algeria and (ii) the player could not re-enter Algeria due to a temporary border closure^86^.

### 4.4 Player injuries

The jurisprudence's position on this matter is clear: a player's injury cannot serve as a valid rationale for either the club to suspend salary payments or for the termination of a contract on the grounds of just cause. In the CAS 2015/A/4003^87^ award, the Panel concluded that a player's injury did not interrupt the club's obligation to pay his salary. Given this reasoning and the fact that the player was owed four months’ salary when he terminated the contract, the panel determined that he had just cause to terminate his contract.

Similarly, in the CAS 2014/A/3509 award, the Sole Arbitrator considered that a clause allowing the contract to be terminated without any compensation and linking this option solely to the passage of time (three months) and a physical injury appeared unfair and oppressive to the player. The application of such a rule was not acceptable because it had a unilateral character and benefited the club exclusively^88^.

Protecting injured players from contract termination is essential to ensure job security and financial stability, especially since injuries are inherent risks in professional football. Without such protections, clubs could unfairly terminate contracts, leaving players without income or proper medical support during their recovery. A strong regulatory framework prevents clubs from exploiting injuries as a reason for dismissal, reinforcing fairness and ensuring that players receive the necessary time and resources to return to full fitness (16).^89^

However, this issue should be explicitly addressed in the regulations to fully protect players from potential abuses by clubs. The rules should not only prevent unjust contract termination due to injury but also establish clear guidelines for cases where an injury results in a permanent or long-term incapacity to play football. A well-defined regulatory framework would ensure fairness for both players and clubs, balancing job security for injured players with reasonable provisions for clubs facing prolonged player absences.

## 5 Conclusion

This article critically examines the evolution of the principle of “just cause” in FIFA's Regulations on the Status and Transfer of Players (RSTP), with a focus on the amendment introduced by the Interim Regulatory Framework. While FIFA's objective is to “provide greater clarity and predictability,” the study finds that the current regulatory framework does not substantially change the existing situation nor address the identified gaps.

The amended Article 14(1) introduces, for the first time, a general definition of “just cause” to guide stakeholders and codify the jurisprudence of FIFA DRC and CAS. This definition reflects FIFA's desire to strengthen case-by-case assessments based on the principles of good faith and reasonableness. However, this formal advancement does not resolve persistent uncertainties regarding the threshold of serious misconduct justifying legitimate contract termination. The vague and unrefined wording of Article 14(2) also creates interpretative uncertainty regarding abusive behavior, thus limiting the consistency of judicial decisions.

Furthermore, in our view, Article 14bis remains rigid, allowing players to unilaterally terminate their contracts in the event of late payment, without distinguishing between fixed salaries and performance bonuses. This automatic approach does not take into account the financial reality of many clubs. This system affects clubs operating in economically fragile markets and contributes to exacerbating financial crises rather than resolving them.

Article 15 remains limited in its application. Indeed, the unclear and restrictive conditions for establishing sporting just cause render the provision ineffective in providing a viable solution to marginalized players. Also, FIFA and CAS jurisprudence exclude poor performance as a reason for contract termination. By doing so, FIFA departs from labor law standards, where dismissal can be justified if based on objective criteria and procedural safeguards. In light of these ambiguities, this study highlights the critical importance of drafting clear and precise contractual clauses, whether individually negotiated by clubs or embedded in standardized contracts issued by leagues.

To balance players’ and clubs’ interests and align with the economic realities of global football, future reforms should aim to:

1. Provide further clarification within Article 14(1) RSTP on the notion of “just cause”, by clearly articulating the key considerations already implicit in the regulatory framework and developed in CAS and DRC jurisprudence, such as the seriousness of the breach, attribution of responsibility, procedural fairness, and proportionality, in order to enhance legal certainty and consistency while maintaining the flexibility of case-by-case assessment.
2. Clarify the notion of abusive behavior justifying termination of the contract for just cause under Article 14(2) by introducing objective criteria drawn from CAS and DRC jurisprudence to reduce interpretative uncertainty and inconsistent application;
3. Provide further clarification within the RSTP regarding the role of prior notice in cases of termination for just cause not covered by Article 14bis, by reflecting the principles developed in the FIFA Commentary and CAS jurisprudence. In particular, this could acknowledge a general expectation that the aggrieved party formally notifies the counterparty and grants an opportunity to remedy the breach, subject to exceptions in cases of particularly serious misconduct, thereby enhancing legal certainty while preserving flexibility.
4. Refine Article 14bis by differentiating between types of remuneration, ensuring that performance-related bonuses are not treated as equivalent to fixed salary obligations, and allowing limited flexibility where clubs face temporary financial constraints;
5. Recognize narrowly tailored contractual provisions addressing severe and sustained underperformance, provided that they are based on objective criteria and subject to robust procedural safeguards;
6. Clarify and modernize the concept of sporting just cause under Article 15, including a more realistic interpretation of the “established player” criterion and the 10% participation threshold, as well as greater transparency in the calculation of compensation.

In practical terms, these reforms would contribute to a more coherent and predictable regulatory framework, capable of reducing legal uncertainty while preserving the core objective of contractual stability. At the same time, they would ensure that the system remains responsive to the economic and professional realities of modern football, thereby achieving a more balanced reconciliation between the protection of players’ rights and the legitimate interests of clubs.

While the Interim Regulatory Framework represents an important step towards codifying and clarifying procedures, further refinements seem necessary to ensure legal certainty, proportionality, fairness, and practical effectiveness. Only a more structured yet flexible approach can guarantee contractual stability and fairness for all stakeholders.

## Data Availability

The original contributions presented in the study are included in the article/Supplementary Material, further inquiries can be directed to the corresponding author.

## Footnotes

^1^For a comprehensive explanation of the protection afforded by FIFA for minors, see Yilmaz, S. Protection of minors: lessons about the FIFA RSTP from the recent Spanish cases at the Court of Arbitration for Sport. *Int Sports Law J* 18, 15–28 (2018). https://doi.org/10.1007/s40318-018-0126-y

^2^FIFA's and other sports regulatory bodies’ functions are now more closely linked to legal frameworks and experts. Those adversely affected by the regulations and rulings of governing bodies are more inclined to contest them in court as long as commercial and economic interests in sports continue to expand. Legal experts now play a more significant role in representing both the regulated parties and the governing bodies themselves, and the regulatory procedures of these organizations have also grown more codified and legal in character. Gardiner, S., Welch, R., Boyes, S., & Naidoo, U. (2011). Sports Law (4th ed.). Routledge. https://doi-org.zulib.idm.oclc.org/10.4324/9780203180884 p.89.

^3^Houben, R. “Sports Governance (in Football) Under Attack.” International Sports Law Journal 23 (2023): 271–292. https://doi-org.zulib.idm.oclc.org/10.1007/s40318-023-00253-6.

^4^See Duval, Antoine, and Ben Van Rompuy, eds. *The Legacy of Bosman: Revisiting the Relationship Between EU Law and Sport*. The Hague: T.M.C. Asser Press, 2016. https://doi.org/10.1007/978-94-6265-120-3.

^5^For more information on the influence of European Union regulations on FIFA regulations, see Christopher A Flanagan, *Who regulates the regulators? How European Union Regulation and Regulatory Institutions May Shape the Regulation of the Football Industry Globally*. in The European Roots of the Lex Sportiva How Europe Rules Global Sport Swedish Studies in European Law Volume 18, 2024. p.119

^6^In its new FIFA Football Agent Regulations, FIFA has capped the service fees that football agents can charge. On October 1, 2023, the corresponding rules went into effect. This decision was widely contested and seems incompatible with EU anti-trust law, see Heermann, P.W. Will the football agents’ service fee cap survive the current legal attacks? *Int Sports Law J* **24**, 188–203 (2024). https://doi.org/10.1007/s40318-024-00279-4.

^7^Case C-650/22 (Lassana Diarra and FIFPRO v. FIFA and URBSFA), 4 October 2024, https://curia.europa.eu/juris/document/document.jsf?docid=290690&mode=req&pageIndex=1&dir=&occ=first&part=1&text=&doclang=EN&cid=12503709

^8^In the Lassana Diarra case, the CJEU addressed the question of whether a new club should be jointly and severally liable for compensation owed by a player to his former club if the player terminates his contract without just cause. The case also raised questions about the basis on which compensation is calculated in such cases, raising concerns about transparency and fairness. In addition, the decision criticized the ability of clubs to challenge the issuance of International Transfer Certificates (ITCs) following a breach of contract, highlighting the need to review these aspects to achieve a better balance in the regulations.

^9^On the solidarity mechanism and its relationship with European Union Law, see Laskowski, J. Solidarity compensation framework in football revisited. Int Sports Law J 18, 150–184 (2019). https://doi.org/10.1007/s40318-018-0134-y

^10^FIFA opens global dialogue on article 17 of the Regulations on the Status and Transfer of Players, October 18, 2024, https://inside.fifa.com/legal/football-regulatory/news/fifa-opens-global-dialogue-on-article-17-of-the-regulations-on-the-status-and-transfer-of-players, the discussions focused on critical aspects such as the criteria for calculating compensation for breach of contract, the sanctions for breach of contract, and the mechanisms for issuing international transfer certificates (ITC)

^11^On December 22, 2024, the FIFA Council Bureau approved the Interim regulatory framework. It entered into effect on January 1, 2025. The Interim regulatory framework introduces significant modifications to key rules and principles. It redefines the method for determining damages owed for breach of contract by either players or coaches. Additionally, it amends the burden and standard of proof concerning joint and several liability in compensatory awards, the evidentiary requirements related to inducement to breach a contract, and the potential sporting sanctions imposed on a player's new club. The framework also clarifies procedural aspects regarding issuing an International Transfer Certificate (ITC). More relevant to our research, the Interim regulatory framework provides a more detailed definition of "just cause". 

^12^FIFA's position on the Lassana Diarra case (27), Interview, with FIFA Chief Legal & Compliance Officer Emilio García Silvero, Press Release October 14, 2024, https://digitalhub.fifa.com/m/3ed083bc1084a958/original/FIFA-s-position-on-the-Lassana-Diarra-case.pdf. According to FIFA, the basic framework of its authority over football contracts remains intact, and the decision does not threaten FIFA's right to regulate international football. The CJEU emphasized the importance of respecting contracts, noting that neither players nor clubs can unilaterally terminate contracts without facing consequences. FIFA views the ruling as a confirmation of the necessity of regulatory safeguards, such as compensation and sanctions, to protect the integrity of sporting competitions and ensure contractual compliance.

^13^Art. 14 Paragraph 1, Regulations on the Status and Transfer of Players incl. Interim regulatory framework January 2025.

^14^FIFA, Explanatory Notes – Interim Regulatory Framework (26), p.6.

^15^The FIFA Commentary on the RSTP infers these principles on the basis of consistent CAS and FIFA DRC jurisprudence, see FIFA, Commentary on the RSTP (21), p.129.

^16^FIFA, Commentary on the RSTP (21), p.107.

^17^CAS 2014/A/3684 Leandro da Silva v. Sport Lisboa e Benfica, CAS 2014/A/3693 Sport Lisboa Benfica v. Leandro da Silva, DRC decision of 19 June 2017, no. 06170253 06170253 E; see FIFA, Commentary on the RSTP (22), p.108.

^18^CAS 2013/A/3091, 3092 et 3093, FC Nantes v. FIFA & Al Nasr Sports Club award of 2 July 2013, para 191, Arbitration CAS 2006/A/1062 Da Nghe Football Club v. Ambroise Alain François Ndzana Etoga, award of 27 July 2006.

^19^CAS 2006/A/1180, as referenced in CAS 2018/A/6050.

^20^DRC decision of 19 November 2020 no. 20-01412 Danilo Pantic, Serbia v. Fehervar FC, Hungary; DRC decision of 22 October 2020 no.20-00912, Patrick Tignyemb, Cameroon v. Bloemfontein Celtic FC, South Africa.

^21^CAS 2015/A/4327 ; CAS 2013/A/3091, 3092& 3093 ; CAS 2013/A/3398. 

^22^CAS 2016/A/4884 FC Ural Sverdlovsk v. Toto Tamuz, FIFA, Commentary on the RSTP, p.108.

^23^CAS 2018/A/6029 Akhisar Belediye Gençlik ve Spor Kulübü Derneği v. Marvin Renato Emnes; FIFA, Commentary on the RSTP (21), p.130.

^24^CAS 2016 / A / 4693 para. 104.

^25^CAS 2015 / A / 4327 ; CAS 2013 / A / 3091, 3092 & 3093 ; CAS 2013 / A / 3398.

^26^See below 3.1.1.3.

^27^TAS 2018/A/5955 et 5981, CAS 2017/A/5465, CAS 2006/A/1180 S 2006 /A/1100.

^28^ DRC decision of 23 January 2013, ° n°0113797 & DRC decision of 10 December 2009 no. 129795 referenced by CAS 2019/A/6533.

^29^DRC decision of 23 March 2023 no. FPSD-7693, Esperance Sp. De Tunis, Tunisie c. Ilyes Cheti, Algérie & Angers SCO, France.

^30^DRC decision of 22 October 2020 no. 20-00807, Aristote Ngondala Fundu, Congo v. Club Academica Clinceni, Romania.

^31^DRC decision of 22 October 2020 no.20-00912, Patrick Tignyemb, Cameroon v. Bloemfontein Celtic FC, South Africa.

^32^FIFA Commentary on the RSTP (22), p. 116.

^33^Arbitration CAS 2017/A/5366 Club Adanaspor v. Mbilla Etame Serges Flavier, award of 7 August 2018.

^34^CAS 2020/A/6854 Wuhan Zall FC v. Jorge Sammir Cruz Campos (Award 26 April 2022), FIFA, CAS & Football Annual Report 2022, p.34.

^35^E.g., UEFA's home-grown player rule imposed limitations on club autonomy and has been legally contested for its potential restriction of free movement under EU law. Regulations aimed at encouraging local talent development may conflict with EU legal principles on the free movement of workers. See Powell, A., Couse, C. Justifying indirectly discriminatory restrictions: can European football's home-grown players rules withstand the proportionality test? Int Sports Law J 23, 436–440 (2023). https://doi.org/10.1007/s40318-024-00262-z; Parrish, R., Živić, L. Royal Antwerp and home-grown players: re-shaping sports governance and EU sports law and policy. Int Sports Law J 23, 453–459 (2023). https://doi-org.zulib.idm.oclc.org/10.1007/s40318-024-00263-y

^36^Stephen F. Ross, Player Restraints and Competition Law throughout the World, 15 Marq. Sports L. Rev. 49 (2005).

^37^The second paragraph of Article 14 RSTP was introduced by FIFA Circular No. 1625 on April 26, 2018 and came into effect on June 1, 2018.

^38^CAS 2015/A/4286 Sebino Plaku v. Wroclawski Klub Sportowy Slask Wroclaw S.A; CAS 2014/A/3642 Erik Salkic v. Football Union of Russia (FUR) & Professional Football Club Arsenal; CAS 2013/A/3398 FC Petrolul Ploiesti v. Aleksandar Stojmirovic; DRC decision of 31 October 2019, Karadzhov; DRC decision of 15 November 2018, no. 11181952-E; FIFA, Commentary on the RSTP (22), p. 109.

^39^FIFA, Commentary on the RSTP (22), p. 111.

^40^FIFA, Commentary on the RSTP (22), p. 109.

^41^CAS 2011/A/2428 I. v. CJSC FC Krylia Sovetov; CAS 2018/A/6029 Akhisar Belediye Gençlik ve Spor Kulübü Derneği v. Marvin Renato Emnes FIFA, Commentary on the RSTP (22), p.110.

^42^CAS 2013/A/3074 Club KS Lechia Gdańsk v. Bedi Buval; FIFA, Commentary on the RSTP (22), p.110.

^43^CAS 2014/A/3643 Club Promotora del Pachuca de C.V. v. Facundo Gabriel Coria & FIFA.

^44^CAS 2013/A/3074 Club KS Lechia Gdańsk v. Bedi Buval

^45^FIFA Commentary on the RSTP (22), p.110.

^46^CAS 2019/A/6171, Josue Filipe Soares Pesqueira v. Osmanlispor FC & CAS 2019/A/6175 Osmanlispor FC v. Josue Filipe Soares Pesqueira & FIFA; FIFA Commentary on the RSTP (22), p.111.

^47^Décision de la CRL du 17 Janvier 2020, Gikiewicz ; FIFA Commentary on the RSTP (22), p.111.

^48^FIFA Commentary on the RSTP (22), p.112.

^49^FIFA Commentaire du Règlement du Statut et du Transfert des Joueurs (25), p.38.

^50^Clubs also include buyout clauses in player contracts, which offer an alternative route for contract dissolution but raise concerns regarding legal enforceability and the integrity of football contracts; see Giancaspro, M. Buy-out clauses in professional football player contracts: questions of legality and integrity. Int Sports Law J 16, 22–36 (2016). https://doi-org.zulib.idm.oclc.org/10.1007/s40318-016-0088-x. Alhendi (20).

^51^Several prominent cases have demonstrated how clubs apply disciplinary clauses in practice. Players have been dismissed for serious misconduct, such as criminal convictions, breaches of team discipline, or violations of substance abuse policies. In some instances, terminations were upheld due to clear contractual breaches, while in others, the procedures followed by clubs were later challenged and found procedurally flawed. These examples highlight the importance of balancing disciplinary enforcement with adherence to fair process; De Marco QC, Nick. 2018. *Football and the Law*. London: Bloomsbury Publishing Plc. Accessed February 18, 2025. ProQuest Ebook Central. p. 103

^52^Football players’ contracts are usually made under standard form contracts drafted by the leagues and aiming at balancing the interests of the league and the clubs on the one hand and the players on the other with respect to the protection of the players under national and European labor laws. For the example of the Bundesliga Standard Player Agreement see. Frodl, C. Neuer, Hummels, Müller, Götze & Co: the legal framework governing industrial relations in German professional football. Int Sports Law J 16, 3–21 (2016). https://doi-org.zulib.idm.oclc.org/10.1007/s40318-016-0094-z

^53^For instance, in the Premier League contract (Clause 10), a club may terminate the employment of a player under specified circumstances. These include cases where the player is guilty of gross misconduct, fails to comply with a final written warning within the disciplinary procedure, or is convicted of a criminal offense for which the sentence is at least three months and not suspended; De Marco QC, Nick. 2018. *Football and the Law*. London: Bloomsbury Publishing Plc. Accessed February 18, 2025. ProQuest Ebook Central. p. 103

^54^Article 18.5 RSTP provides that « If a professional enters into more than one contract covering the same period, the provisions set forth in Chapter IV shall apply. ».

^55^CAS 2020/A/6796 Andriamirado Aro Hasina Andrianamimanana & Kaizer Chiefs FC v. Fosa Juniors FC & Fédération Internationale de Football Association (FIFA), award of 27 October 2020, DRC decision of 8 October 2020 no. 20-00247, TP Mazembe, Congo DR v. Meschack Elia Lina, Congo DR & BSC Young Boys, Suisse, DRC decision of 8 October 2020 no. 20-00760 Saham Club, Oman V. Guibihi Florent Didier Kore, Côte d'Ivoire & Naft Al Wasat SC, Iraq.

^56^FIFA, Commentaire du Règlement du Statut et du Transfert des Joueurs (2007), p.55.

^57^Article 14bis RSTP par. 1 provides that: “In the case of a club unlawfully failing to pay a player at least two monthly salaries on their due dates, the player will be deemed to have a just cause to terminate his contract, provided that he has put the debtor club in default in writing and has granted a deadline of at least 15 days for the debtor club to fully comply with its financial obligation(s). Alternative provisions in contracts existing at the time of this provision coming into force may be considered”.

^58^FIFA, Commentary on the RSTP (Ed. 2021), p.121.

^59^CAS 2020/A/7093 Tractor Sazi Tabriz FC v. Anthony Christopher Stokes & Adana Demirspor KD.

^60^DRC decision 28 February 2020, Pablo. DRC decision 23 April 2020, Junuzovic ; DRC decision 23 April 2020, Cueva Bravo ; DRC decision 10 December 2020, Markoski; De Weger L and Al A, « Termination of employment contracts due to outstanding salaries : Overview of FIFA jurisprudence under Article 14bis FIFA RSTP », Football Legal, 30 June 2021, https://www.football-legal.com/content/termination-of-employment-contracts-due-to-outstanding-salariesoverview-of-fifa-jurisprudence-under-article-14bis-fifa-rstp.

^61^DRC decision of 10 December 2020, Markoski; De Weger F and Al A.

^62^With regard to bonuses, a CAS award clarified that a bonus can only be regarded as part of a player's salary if its amount has been explicitly fixed or can be objectively determined. When this condition is met, the bonus qualifies as an integral component of the salary, CAS 2020/A/7262 Helder Jorge Leal Rodrigues Barbosa & Hatayaspor CA v. Akhisar Belediye Genclick ve SK , Commentary on the RSTP (21) p. 153.

^63^DRC decision of 7 June 2018, no. 06181022-E, FIFA Commentary on the RSTP (22), p. 129.

^64^Parrish, R., & Pendlebury, A. (2018). Football law. Routledge Handbook of Football Business and Management (pp. 71-87). Routledge. https://doi-org.zulib.idm.oclc.org/10.4324/9781351262804-7. Alhendi et al. (19).

^65^RSTP (2023), Art. 5.1.

^66^FIFA Commentary on the RSTP (22), pp.114 & 115.

^67^TAS 2016/A/4509 Espérance sportive de Tunis c. Moussa Marega, CAS Bulletin 2017/1 p.76. 

^68^Arbitration CAS 2014/A/3643 Club Promotora del Pachuca S.A. de C.V. v. Facundo Gabriel Coria & Fédération Internationale de Football Association (FIFA), award of 5 June 2015.

^69^Arbitration CAS 2015/A/4122 Al Shaab FC v. Aymard Guirie, award of 26 August 2016.

^70^According to Article 17(4) of the FIFA RSTP, a contract's validity cannot be conditioned on a successful medical examination or the issuance of a work permit. This imposes a duty on clubs to conduct all necessary medical assessments of a player's health and physical condition before finalizing an employment contract. Failure to do so may be considered negligence on the club's part. If a contract includes a suspensive condition requiring a medical examination, the contract remains valid even without this clause. Should the club refuse to honor the contract on these grounds, it would be held responsible for its termination without just cause. Moreover, a club cannot justify withdrawing from a contract due to a player's injury, whether actual or merely suspected. Łukomski, J. On the finalisation of international football transfers and professional football players’ contracts. *Int Sports Law J* 20, 157–179 (2020). https://doi-org.zulib.idm.oclc.org/10.1007/s40318-020-00170-y

^71^Several decisions are mentioned by the FIFA Commentary on RSTP, notably : DRC decision of 25 February 2020, Elisee, DRC decision of 19 February 2020, Bukari, DRC decision of 15 November 2018, no. 11180788-E, CAS 2017/A/5164 FAT v. Victor Jacobus Hermans, CAS 2017/A/5092 Club Hajer FC Al-Hasa v. Arsid Kruja.

^72^FIFA Commentary on the RSTP (22), p. 115.

^73^DRC Decision of 4 June 2020 n°00471-20, Club Athlétique Bizertin v. Boubakar Traore para. 18.

^74^DRC decision of 15 July 2020 n°00470-20, Miroslav v. Police Benglasdesh.

^75^DRC decision of 19 November 2020 n° 20-01412, Danilo Pantic, Serbia v. Fehervar FC, Hungary para. 19 et 20.

^76^DRC decision of 22 October 2020, no. 20-01164, Gafar Adefolarin Durisnmi, Nigeria Trat FC, Thailand.

^77^DRC decision of 11 April 2019, no. 04190658-E.

^78^DRC decision of 22 October 2020, no. 20-01073 Muhindo Kirenge Piscas, Congo DR v. Wazito FC, Kenya, DRC decision of 22 October 2020, no. 20-01075, Paul Acquah, Ghana v. Wazito FC, Kenya.

^79^DRC decision of 13 February 2020, Advic, DRC decision of 29 January 2020, Boskovic, CAS 2016/A/4846 Amazulu FC v. Jacob Nambandi & FIFA & National Soccer League, CAS 2018/A/6041 Theofanis Gekas v. Akhisar Belediye Gençlik, FIFA Commentary on the RSTP (22), p.113.

^80^CAS 2015/A/4042 Gabriel Fernando Atz v. PFC Chernomorets Burgas, DRC decision of 11 January 2023, (Santos Gonzaga); DRC decision of 8 March 2023 (Milutinovic); Commentary on the RSTP 2013, p. 135.

^81^For instance, under UK labor law, poor performance is considered a valid reason for dismissal if the employer has issued warnings and offered improvement opportunities Alhendi and Baniamer (18).

^82^DRC decision of 22 October 2020, no. 20-01073 Muhindo Kirenge Piscas, Congo DR v. Wazito FC, Kenya, DRC decision of 22 October 2020, no. 20-01075, Paul Acquah, Ghana v. Wazito FC, Kenya.

^83^FIFA, Circular n°1720 of 11 June 2020 (24).

^84^DRC decision of 22 October 2020, no. 20-01073 Muhindo Kirenge Piscas, Congo DR c. Wazito FC, Kenya, DRC decision of 13 November 2020 no. 20-01218, Raul Andrei Rusescu, Romania v. Giresunspor Kulubu Dernegi, Turkey.

^85^CAS 2021/A/8118 Anorthosis Famagusta FC v. Ruben Rayos Serna (Award 28 March 2022), CAS 2021/A/7878 & 7916 Naim Sliti v. Al Ettifaq Club (Award 27 April 2022), CAS 2021/A/8216 Besiktas AS v. Loris Sven Karius (Award 24 January 2022), FIFA, CAS & Football Annual Report 2022 (23), p. 32.

^86^CAS 2021/A/7851 & 7905 Mohamed Naoufel Khacef & Tondela Futebol v. FIFA (Award 11 January 2022), FIFA, CAS & Football Annual Report 2022 (23), p.40.

^87^CAS 2015/A/4003 Maccabi Haifa v. Anderson Conceicao Xavier & Clube de Regatas Vasco da Gama, FIFA Commentary on the RSTP (22), p.113. Al-Hendi (17).

^88^CAS 2014/A/3509 Anorthosis Famagusta FC v. Wojciech Kowalewski, award of 25 September 2014.

^89^On how players can be pressured to play, see Law, G., & Bloyce, D. (2017). ‘Pressure to play?’ A sociological analysis of professional football managers’ behaviour towards injured players. Soccer & Society, 20(3), 387–407. https://doi-org.zulib.idm.oclc.org/10.1080/14660970.2017.1321540
